# New Quarters in Regent's Park

**Published:** 1920-01-17

**Authors:** 


### THE WEST END HOSPITAL FOR NERVOUS DISEASES.

### New Quarters in Regent's Park.

About the middle of November the in-patient department of this hospital for diseases of the nervous system was moved from its well-known quarters in Welbeck Street to the newly-acquired premises in Regent's Park. Such a move on the part of the hospital, especially as it contained bedfast patients, must have called for very careful organisation and forethought, as well as for hard work on the part of the staff; it was much facilitated by the use of an ambulance lent for the purpose by the hospital's neighbours, Messrs. Debenham and Freebody. The move was successfully accomplished, and the work of the in-patient department continued without intermission. By the end of November it was noticed already that the general health and appearance both of patients and staff had benefited by the change to airier surroundings.

The West End Hospital was established in 1878; by 1910 it had equipped and opened thirty-seven beds for in-patients. During the war the influx of military cases and the opening up of various temporary premises scattered about the neighbourhood (including Bulstrode Street) resulted in a rise of bed strength to one hundred. The premises now opened in Regent's Park contain accommodation for seventy-one patients, and their opening renders possible great expansion and improvement of the work of the out-patient department at 73 Welbeck Street. The Bulstrode Street premises, at one time used for patients, and more recently as a nurses' home, are now closed, and are on the market.

The new in-patient department is on the east side of Regent's Park, about halfway between the Zoological Gardens and the Botanical Society's Gardens, and only a short walk from each; indeed, it is said that the lions, tigers, and other animals are audible in the wards in the early morning, before London's noises drown their cries. It occupies the premises known as St. Catherine's Lodge, opposite St. Catherine's Church and College. The latter foundation dates from very early times, for it is said to have been originally endowed by the wife of King Stephen. When the old site was acquired for the making of St. Catherine's Docks a move was made to Regent's Park; and St. Catherine's Lodge was built to house one of the chief administrators of the Society—this was in 1828. An extra wing has since been built to the house, which stands in five acres of grounds. During the latter part of the war it was converted into an American Red Cross Hospital for officers by the generosity of Mr. Salomon, from whom the remaining Crown lease of nine years has been acquired by the West End Hospital on terms which are exceedingly generous. The result is that the hospital moves into buildings which were, admittedly, not designed for institutional purposes, but for a mansion. On the other hand, many alterations and improvements for hospital purposes were made by the owner when he converted it to Red Cross use; so this drawback is minimised to a considerable extent. For the purpose of treating nerve cases small wards are no detriment, so that, on the whole, the house is very fairly suitable to its new functions. It is entirely surrounded by its own grounds, and the environment of the Park ensures both quiet and privacy, as well as abundance of light and air. On the occasion of our visit the Matron, Miss C. Thorpe, R.R.C., very kindly showed us the whole of the premises; for as yet there is no resident medical officer appointed, though accommodation has been reserved for one in due time. We were also privileged to meet the Treasurer, General Willoughby, and the Secretary, Mr. D. D. Kirkaldy.

Much of the equipment has been taken over from Mr. Salomon, and many of the fittings are of almost a luxurious kind for that reason. Central heating is installed throughout, though one or two of the largest rooms have fireplaces as well. On the ground floor, besides two men's wards, the most noticeable features are a fine day-room for male patients, a board-room, and a splendid children's ward. The latter is easily the best room in the house (it looks as if it were designed originally as a ballroom), and a large French window opening straight on to a delightfully secluded part of the grounds should be a great asset to the patients in summer, when they can be moved outside for most of the day.

Upstairs on the first floor are the surgical wards and the women's wards, together with the operating suite. Here also are two wards for paying patients: one charming ward of four beds for those who can pay four guineas a week or thereabouts, and one for those who can pay a guinea a week. The latter ward has four beds in it, but has barely room for three, and is badly overcrowded with four. The wards in general are very pleasant and comfortable: their sanitary annexes are mostly both insufficient and unsatisfactory in other respects—*e.g.* bath and w.c. in the same apartment—but this is only to be expected in a building not designed for the large population which it now contains.

The operating-theatre has very poor natural illumination, but is well lit by indirect artificial light. The anæsthetising room is across a passage from the theatre, which is not an ideal arrangement, but unavoidable in the circumstances.

The top floor contains the nurses' bedrooms, located in attics. Several of these small rooms contain three beds each, a very serious overcrowding, as well as objectionable from want of privacy. The arrangement is perhaps necessary at the moment, for there are no buildings anywhere near which could be turned into a nurses' home; but steps should be taken to alter it at the earliest possible moment. These rooms could then be allocated to the domestic staff, at present provided for in the basement. The shortness of the lease-remainder (nine years), and the fact that the Crown is the landlord (notoriously more "difficult" to deal with than private owners are) will naturally deter the Committee at present from building a home, for which there is ample room in the grounds; but we are glad to hear that the managers are fully aware of the inadequate accommodation at present offered to their nurses, and have every intention of overcoming the defect as soon as possible. An eight-hour (three-shift) day is being worked.

Of the auxiliary departments, a laundry is in process of installation in a suitable room at the north end of the buildings. Its establishment should result in substantial economies of working expenses. There is at present no pathological laboratory and no mortuary; both these are definite wants, to be filled as and when the financial situation admits. There is also no isolation ward—always a need wherever children are admitted. Fortunately there is plenty of space available for building operations when they become feasible, but all such plans are presumably in abeyance until some tenure of the property more substantial than the existing short lease shall be secured.

